# Genetic exchange and reassignment in *Porphyromonas gingivalis*

**DOI:** 10.1080/20002297.2018.1457373

**Published:** 2018-04-12

**Authors:** Ingar Olsen, Tsute Chen, Gena D Tribble

**Affiliations:** a Department of Oral Biology, Faculty of Dentistry, University of Oslo, Oslo, Norway; b Department of Microbiology, Forsyth Institute, Cambridge, MA, USA; c Department of Oral Medicine, Infection, and Immunity, Harvard School of Dental Medicine, Boston, MA, USA; d Department of Periodontics and Dental Hygiene, School of Dentistry, University of Texas Health Science Center at Houston, Houston, TX, USA

**Keywords:** *Porphyromonas gingivalis*, adaptability, survival, periodontitis, keystone pathogen hypothesis, homeostasis, dysbiosis

## Abstract

*Porphyromonas gingivalis* is considered a keystone pathogen in adult periodontitis but has also been associated with systemic diseases. It has a myriad of virulence factors that differ between strains. Genetic exchange and intracellular genome rearrangements may be responsible for the variability in the virulence of *P. gingivalis*. The present review discusses how the exchange of alleles can convert this bacterium from commensalistic to pathogenic and potentially shapes the host-microbe environment from homeostasis to dysbiosis. It is likely that genotypes of *P. gingivalis* with increased pathogenic adaptations may spread in the human population with features acquired from a common pool of alleles. The exact molecular mechanisms that trigger this exchange are so far unknown but they may be elicited by environmental pressure.

## Introduction

*Porphyromonas gingivalis* is considered a keystone pathogen in periodontitis [1] but it may also be involved in several periodontitis-related systemic diseases [2]. When the prevalence of periodontitis was estimated in Americans >65 years, almost two-thirds (62.3%) had one or more periodontal sites with ≥5 mm of clinical attachment loss and almost 50% had at least one site with probing pocket depth of ≥4 mm [3]. The older adult population is growing rapidly in the Western world and it is estimated that by 2040 the number of adults ≥65 years will be about 50% in the USA [3]. Their oral health status will be very important in order to maintain an adequate quality of life.

*P. gingivalis* expresses a myriad of virulence factors [4]. Since genetic exchange may cause diversity in specific virulence factors in *P. gingivalis*, the present review will give an overview on how genetic exchange and rearrangement may occur in this anaerobic bacterium.

### Natural competence and conjugation

It is well known that prokaryotes can respond dynamically to changing environments by genetic exchange [5]. *P. gingivalis* exchanges chromosomal DNA between strains by natural competence and conjugation [6–8]. By using antibiotic resistance markers, it was demonstrated that the strains ATCC 33277^T^ and W83 of *P. gingivalis* were able to exchange chromosomal DNA resulting in offspring, which carried DNA from both donor and recipient [6]. The chimeras thereby created had altered phenotypic features as demonstrated through biofilm formation [9]. Natural competence affected by the competence protein ComF was found to be the predominant form of DNA transfer in *P. gingivalis* while transfer by a conjugation-like approach occurred at a lower frequency [6]. Subsequently, natural competence mechanisms have been detected in multiple *P. gingivalis* strains and the DNA uptake is not sensitive to the DNA source or modification status [6]. The major source for horizontal DNA transfer and allelic exchange between strains was found to be extracellular DNA present in *P. gingivalis* biofilms. Moreover, *P. gingivalis* may even scavenge DNA from other species as a nutritional source [6].

A high level of reticulation was seen in the phylogenetic networks constituted by 23 strains of *P. gingivalis* [8]. This indicated extensive horizontal gene transfer between the strains. There were variants in the major virulence factor of the Kgp proteinase, the Kgp C-terminal cleaved adhesion domain and the surface proteins FimA, FimCDE, Mfa l, RagAB, Tpr and PrtT. It was concluded that *P. gingivalis* uses domain rearrangements and genetic exchange to generate diversity in specific surface virulence factors. When the whole-genome sequences between ATCC 33277 and W83 were determined, extensive rearrangements were found between the two strains, possibly induced by different mobile elements (insertion sequences, miniature inverted-repeat transposable elements, and conjugative transposons) [10]. In ATCC 33277, many of the genes showed higher similarity to the genes of other bacterial species such as *Bacteroides fragilis* than to genes in W83. This suggested that they had been introduced to ATCC 33277 by horizontal gene transfer [10].

The specific combination of genes in the genome of the dominant *P. gingivalis* strain may vary with the disease state and the host response. Probably exchange of DNA in biofilms by transformation is important to the ecology and persistence of *P. gingivalis* in the challenging environment of the periodontal pocket and to evasion of host immune defences and immunization strategies [6,9]. *P. gingivalis* appears to have both a core genome [7,11] and a flexible, dispensable genome. Sampling of DNA from the flexible genome could imply adaptation for the bacterium to the changing environment [7], which is supported by the genetic diversity of *P. gingivalis.*

### Fimbriae types provide selective advantage in periodontal disease

Although genetic variations can be seen in many virulence factors of *P. gingivalis*, most interest has been devoted to the major fimbrial protein FimA [12–14]. The gene encoding the protein subunit that is assembled to form the surface fimbriae of *P. gingivalis* is the *fimA* gene. Six alleles are found in the *fimA* gene: types I–V and Ib. Type II and IV *fimA* have significantly greater adhesive and invasive capabilities and are associated with more cytotoxic strains compared to other *fimA* type clones [15]. While type Ib, II and IV are most frequently associated with periodontitis [14–18], type I and III are less inflammatory and are associated with strains more likely to form localized self-limiting infections [19]. It has been suggested that the *fimA* type II allele confers a selective advantage in the advanced disease process thus ensuring its distribution throughout the *P. gingivalis* population [20].

Another type of fimbria expressed by *P. gingivalis* is Mfa 1. These minor or short fimbriae are mainly composed of polymers of the Mfa 1 protein and are encoded by the *mfa 1* gene [13]. Both FimA and Mfa 1 fimbriae enable bacteria to adhere to surfaces, and are important in biofilm formation [21]. A genetic association between the *fimA* genotype and the presence of both the *mfa 1* and the *53k* gene has been suggested [22]. The *53k* gene encodes the 53K protein (53-kDa major outer membrane protein) of *P. gingivalis*. This gene was located at a locus that corresponded to the *mfa 1* gene [22]. The outer membrane protein has been reported as a major fimbrilin of a novel-type fimbrium [22]. Generally, *P. gingivalis* expresses either 53K or Mfa 1 fimbriae in addition to FimA fimbriae [22]. However, strains with *fimA* genotypes III and V express 53K fimbriae almost exclusively, while *fimA* genotypes I and II tend to express Mfa 1 fimbriae. The downstream gene product Mfa 2 has a role as anchor for the Mfa 1 fimbriae and as regulator of the Mfa 1 filament length [23]. It is likely that the distinct short fimbria-molecules found in different strains as well as long fimbriae both influence initiation and progression of periodontal disease [24].

**Figure 1.**
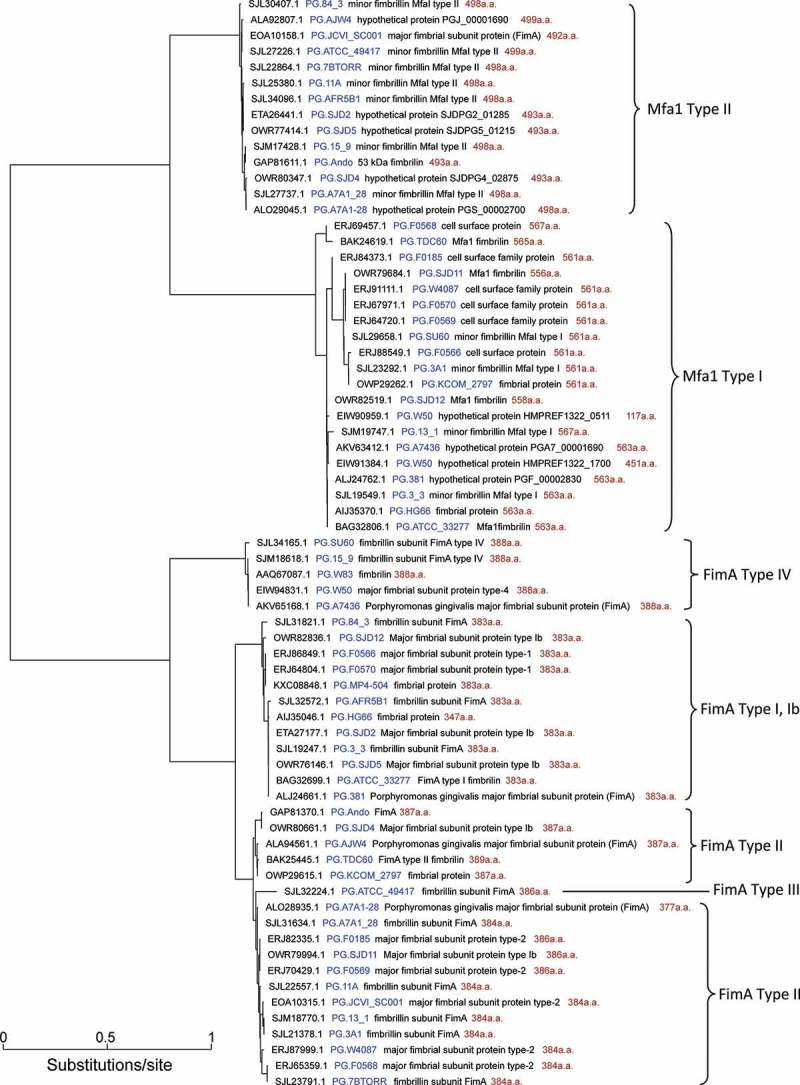
Major and minor fimbrilin proteins encoded in the 35 P. *gingivalis* genomic sequences. All the fimbrilin proteins were detected in the following manner: The keyword FimA/Mfa1/fimbrilin were used to select from the proteins predicted in the 35 genomes, which were annotated by the NCBI prokaryotic genome annotation pipeline [54]. The keywords were also used to search for P. *gingivalis* FimA from the NCBI protein databases. These “seed” proteins were then searched against all the proteins in the 35 genomes to identify additional fimbrilin candidates. These potential candidates matched with the seed proteins at ≥ 90% sequence identity and ≥ 90% length coverage. The additional candidates were combined with other annotated fimbrilin proteins and aligned with the “mafftℍ program [55]. The aligned sequences were used to build a phylogenetic tree with the “FastTree” program [56]. For each fimbrilin shown in the tree, the Genbank accession number was printed first, followed with the strain label (in blue), the original functional annotation (by NCBI), and the size of the protein sequences in amino acids (red).

A survey of the fimbrilin encoding genes among the 35 currently available *P. gingivalis* genomic sequences revealed that most of the *P. gingivalis* strains possess both FimA and Mfa 1 fimbrilin encoding genes (Table 1). The only exceptions were strain MP4-504 and W83, of which only the FimA fimbrilin encoding gene was found in their genomes. Interestingly, strain W50 has been shown to be very similar to W83. However, the Mfa 1 encoding gene was only detected in W50, even though the sequence is unfinished. The number of genomes encoding type I, type Ib, type II, type III and type IV FimA were 9, 3, 17, 1 and 5, respectively. The number of genomes encoding type I and type II Mfa 1 were 19 and 14, respectively. The only genome encoding the Type III FimA gene was the strain ATCC 49417 with the protein sequence ID SJL32224.1 (Figure 1). SJL32224.1 is identical to a previously cloned and derived Type III FimA sequence (Genbank Accession: AHG31000.1) from the same strain [7], thus confirming the presence of this type of FimA in this strain. There are only two other *P. gingivalis* Type III FimA sequences in the NCBI Protein Database – BAA04627 and Q51826, representing a slightly different version of Type III FimA but so far has only been found in strain 6/26 [25].

**Table 1.** Occurrences of transposase, fimbrilin and CRISPR features in 35 *P. gingivalis* genomes*.

		Fimbrilin types		CRISPR features			
Strains	Transposase ^a^	FimA type ^b^	Mfa1 type^c^	CAS ^d^	CRISPR ^e^		Max. DR ^f^
11A	26	II ^h^	II	6		3	14
13_1	17	II ^h^	I	19		5	93
15_9	18	IV	II	15		6	22
381	90	I ^h^	I ^b^	14		3	120
3A1	34	II	I	15		5	67
3_3	20	I ^h^	I	15		5	52
7BTORR	21	II ^h^	II	6		4	15
84_3	25	I ^h^	II	6		2	82
A7436	103	IV ^h^	I	15		5	17
A7A1-28	28	II ^h^	II ^h^	14		4	64
A7A1_28	17	II ^h^	II	15		4	64
AFR5B1	18	I ^h^	II	6		3	67
AJW4	87	II ^h^	II ^h^	1		2	12
ATCC_33277	96	I	I ^h^	14		3	120
ATCC_49417	28	III ^h^	II	16		3	8
Ando	8	II ^h^	II ^h^	2		3	16
F0185	8	II	I ^h^	6		15	13
F0566	17	I	I ^h^	12		7	19
F0568	14	II	I ^h^	6		7	35
F0569	15	II	I ^h^	14		22	13
F0570	11	I	I ^h^	7		3	9
HG66	81	I ^h^	I ^h^	14		3	97
JCVI_SC001	23	II	II ^h^	0		3	6
KCOM_2797	32	II ^h^	I ^h^	17		3	74
MP4-504	35	I ^h^		6		3	37
SJD11	14	II ^j^	I ^h^	13		3	46
SJD12	12	I ^b^	I ^h^	16		5	20
SJD2	10	I ^b^	II ^h^	4		3	22
SJD4	10	II ^i^	II ^h^	15		6	72
SJD5	8	I ^b^	II ^h^	4		3	22
SU60	23	IV	I	15		3	82
TDC60	40	II	I ^h^	15		5	66
W4087	13	II	I ^h^	6		4	19
W50	25	IV	I ^g,h^	15		5	24
W83	59	IV ^h^		15		4	24

^a^ To identify more transposases, sequences of *P. gingivalis* transposases that were annotated in the 35 genomes, as well as additional ones found in the NCBI protein database, were used to search against all the proteins annotated in the 35 genomes. Proteins that were not previously annotated as transposases but had ≥90% identity and ≥90% length coverage to the queries were considered additional transposases.

^b^ To identify more FimA proteins, sequences of *P. gingivalis* FimA that were annotated in the 35 genomes, as well as additional ones found in the NCBI protein database, were used to search against all the proteins annotated in the 35 genomes. Proteins that were not previously annotated as FimA but with ≥90% identity and ≥90% length coverage to the queries were considered additional FimA.

^c^ To identify more Mfa1 proteins, sequences of *P. gingivalis* Mfa1 that were annotated in the 35 genomes, as well as additional ones found in the NCBI protein database, were used to search against all the proteins annotated in the 35 genomes. Proteins that were not previously annotated as Mfa1 but with ≥90% identity and ≥90% length coverage to the queries were considered additional Mfa1.

^d^ CAS: CRISPR Associated System proteins. To identify more CAS proteins, sequences of *P. gingivalis* CAS that were annotated in the 35 genomes, as well as additional ones found in the NCBI protein database, were used to search against all the proteins annotated in the 35 genomes. Proteins that were not previously annotated as Mfa1 but with ≥90% identity and ≥90% length coverage to the queries were considered additional CRISPR-CAS proteins.

^e^ CRISPR: these are the number of CRISPR DNA sequences identified by the online CRISPRfinder programme (http://crispr.i2bc.paris-saclay.fr/Server/) [57].

^f^ Max. DR: Maximal number of direct repeats among the CRISPR identified.

^g^ A full version of Type I Mfa1 in W50 was 451 a.a. in Figure 1.

^h^ These annotations were inferred based on the phylogenetic tree in Figure 1; the original NCBI annotation was not associated with fimbrilin or no fimbrilin type inferred.

^i^ FimA protein of SJD4 was annotated as Type Ib in Figure 1.

^j^ FimA protein SJD11 was annotated as Type Ib in Figure 1.

### Exchange of allele types in fimbriae

Kerr et al. [7] reported that *P. gingivalis* exchanged fimbriae allele types I and IV into four distinct strain backgrounds via natural competence. A complete exchange of the entire *fimA* allele was detected but the rate of exchange varied between different strain backgrounds. Also, genetic exchange within other regions of the genetic locus of fimbriae was seen. Exchange of fimbriae allele type caused marked phenotypic changes, e.g. in the quantity of fimbriae produced, membrane blebbing and autoaggregation. When the type I allele was replaced with the type III or IV allele, increased invasion of gingival fibroblasts were seen compared to that achieved with the isogenic parent strain. By using plasmid vectors, Kato et al. [26] substituted *fim*A of ATCC 33277 with type II *fim*A, and that of OMZ314 (type II strain) with type I *fim*A. Replacement of type I *fim*A with type II increased bacterial adhesion/invasion to epithelial cells, whereas substitution with type I *fim*A resulted in reduced efficiency. Similarly, Kerr et al. [7] found that substitution of the type IV allele from strain W83 into ATCC 33277 transformed the 33277 strain into a strain phenotypically more similar to the W83 strain. Such strategies may be used by pathogenic strains to modify their interactions with the host and thereby change the homeostatic balance in their favour.

### Clonality

The expression of heterogenic virulence properties in *P. gingivalis* may depend on clonal diversity. In clonal populations of bacteria, genetic diversity primarily arises by point mutations. Point mutations within open reading frames that are limited in size can occur in *P. gingivalis* at frequencies of 10^–9^, similar to other bacterial species such as *Escherichia coli* [20].

In non-clonal bacterial populations, however, there is a spectrum of phenotypes from commensal to pathogenic phenotype [27] and a number of allele combinations that can be related to disease [20]. Some allele combinations may be more virulent than others and it is reasonable to think that allele combinations of *P. gingivalis* differ in health and disease. Non-clonal pathogens are common in bacteria associated with chronic diseases. Based on the *P. gingivalis* MLST (Multi Locus Sequence Typing) database (https://pubmlst.org/pgingivalis/) the index (*I_A_*) for estimating the degree of association between alleles at different loci for representative sequence types, was calculated to be 0.3704 suggesting a weakly clonal population structure [15,28]. By using the standardized index of association (*I^S^_A_*), which takes into account the number of genes investigated, thereby providing a better estimate of the linkage equilibrium than does *I_A_*, the value was 0.062. Clonal populations give a score of one, while mono-clonal populations yield zero. This supported a study by Frandsen et al. [29] who found that *P. gingivalis* has a non-clonal population structure with frequent recombination. These figures also yielded support to the idea of *P. gingivalis* consisting mainly of mono-clonal strains established through bacterial transfer of genes *in vivo*, so-called bacterial sex [30]. Consequently, the genetic variation in *P. gingivalis* strains is considerable suggesting that exchange of genes occurs to improve the chances of *P. gingivalis* to survive *in vivo*. This variability can be extensive and comprise entire genes as well as operons [20].

### Pathogenicity islands

Many of the features suggesting lateral gene transfer in *P. gingivalis* are demonstrated through the presence of pathogenicity islands, so called because they contain assemblies of genes for virulence that cause disease [31]. Ranging in size from 10 to 200 kb, pathogenicity islands often carry genes that encode integrases and transposases involved in DNA mobility, and they can be associated with tRNA genes, which are favoured sites for integration of foreign DNA [32]. This was demonstrated by Chen et al. [32] when comparative whole-genome analysis of virulent (W83) and avirulent (ATCC 33277) strains of *P. gingivalis* was made. In W83, one cluster of genes was associated with two paralogous regions of chromosomes with low G + C content that contained conserved and species-specific hypothetical genes, transposons, insertion sequences and integrases and was located close to tRNA genes, having many of the characteristics of pathogenicity islands acquired by lateral gene transfer.

### Rag locus

One pathogenicity island in *P. gingivalis* is the *rag* locus [33]. The *rag* locus of strain W50 encodes *RagA* (*receptor antigen gene A*), a predicted TonB-dependent receptor protein, and *RagB*, a lipoprotein that constitutes an immunodominant outer membrane antigen [33–35]. They probably act together on the surface of the organisms to help active transport, mediated through the periplasmic protein TonB or constitute part of a signal transduction system. The *rag* locus of *P. gingivalis* may have arisen via horizontal gene transfer from *Bacteroides* [33,36]. Supporting that horizontal gene transfer had taken place were the low G + C content of the locus, the association with motility elements and a restricted distribution in the species [34]. In *P. gingivalis*, the exogenous *rag* locus promoted expression of virulence genes or activation of dormant genes [35].

PCR demonstrated that rag+ *P. gingivalis* strains were more frequently present in deep periodontal pockets than in shallow pockets of patients with periodontitis [33,35]. While highly virulent *P. gingivalis* strains always carried the *rag* locus, avirulent strains did not. In mice, *rag-1* isolates more likely caused disease than other rag alleles (*rag-2–4*) [34]. The *rag* locus was also seen in a small portion of sites where *P. gingivalis* was not detected [36], and Frandsen et al. [29] found a random distribution of the *ragB* locus in 132 *P. gingivalis* strains.

### Reca and pepo housekeeping genes

When MLST was performed of clinical *P. gingivalis* isolates from 15 subjects with ‘refractory’ periodontitis, not one but several sequence types were detected for most individual periodontal pockets. The allelic variation in sequence types indicated recombination of the *recA* and *pepO* housekeeping genes in diseased sites [37]. This could have been the result of recombination between different clones of *P. gingivalis* within the periodontal pocket.

### Conjugative transposons and transposons

Conjugative transposons (Ctns) represent a subgroup of integrative and conjugative elements characterized by their ability to move from one bacteria to another through a process requiring cell-cell contact [38]. They can excise themselves from the genome where they are integrated, transfer themselves to a recipient by conjugation and integrate into the recipient genome [39]. Thus CtnPg1, which was the first complete Ctn found in *P. gingivalis*, was able to excise itself from chromosomal DNA using an integrase (Int; PGN-0094) encoded in CtnPg1 and produce a circular intermediate [10]. The transfer of CtnPg1 requires a RecA function in the recipient cell. It carried a complete set of genes for conjugative transfer and integration. When CtnPg1 was transferred from ATCC 33277 to W83, chromosomal gene transfer occurred in addition to the CtnPg1 transfer itself.

Many Ctns carry genes for antibiotic resistance, providing e.g. tetracycline resistance and erythromycin resistance [40] and transfer of Ctns often contributes to spread of antibiotic resistance genes. Ctns have been studied most frequently in *Bacteroides* where many species have large self-transmissible Ctns. ATCC 33277 of *P. gingivalis* carried four copies of Ctns that were not present in W83 [10]. Several genes were moderately homogenous to the sequences of genes of Ctns from *Bacteroides* species. However, the sequences of two copies of novel composite transposons detected in the ATCC 33777 genome were identical [10]. Thus the CtnPg1 family differs from the *Bacteroides* Ctn family and also from the Tn*916*/Tn*1545* family. The CtnPg1 family is distributed among both oral and intestinal bacteria where it can play an important role in horizontal gene transfer, e.g. of antibiotic resistance genes to anaerobic bacteria.

Table 1 shows the number of complete and partial proteins that are homologous to the transposases, ranging from 8 (strains Ando, F0185, and SDJ5) to 103 (strain A7436). However, the lower numbers are from unfinished draft sequences and are likely under-estimated. In general, *P. gingivalis* is a species with a high number of transposases, hence the genetic exchange and genomic locus rearrangement in and between strains are expected to be frequent.

### Crispr-Cas

The clustered regularly interspaced short palindromic repeats (CRISPR) and their associated proteins (Cas) are adaptive immune systems in prokaryotes present in most *Bacteria* and *Archaea*. They provide adaptive immunity against foreign elements such as bacteriophages/viruses, plasmids and transposons. At least 95% of clinical strains from *P. gingivalis* harbours CRISPR arrays [41]. The presence of transposases and CRISPR-associated sequences in *P. gingivalis* [11] suggested that some parts of the *P. gingivalis* genome are mobile so that rearrangement or horizontal gene transfer from one strain to another can take place [20]. In the *P. gingivalis* W83 genome, all four CRISPR regions were transcribed and one of them was active against dsDNA *in vivo* [41]. Probably the CRISPR-Cas system has developed as a protective system that verifies and selects DNA entering bacterial cells, helping them to regulate horizontal gene transfer [41].

Table 1 summarizes the occurrence of the CRISPR-Cas proteins and CRISPR elements detected in the genomes of 35 *P. gingivalis* strains. Strain 13_1 encodes 19 Cas homologous proteins and thus may contain at least 3 CRISPR systems (a Cas operon contains between 6 to 8 ORFs). On the other hand, JSVI SC001, an environmental strain, contains no detectable Cas proteins, even though 3 CRISPR-like DNA sequence structures were detected. The number of CRISPR systems in the genome may reflect the difficulty of incorporating foreign DNA while the number of direct repeats (DR) detected in the CRISPR elements (last column in Table 1) may indicate the CRISPR activity in the past for the genome and how often the strain had encountered the invasion of foreign DNA.

### Proteins and whole genome nucleotide sequences

Protein coding sequences for the virulent W83 strain comprised several genes that may be associated with higher virulence of the strain, including glycosyltransferase, a protein required for capsular polysaccharide biosynthesis, sensor histidine kinase, surface antigen and thiol protease [10].

Phylogenomic comparison based on shared proteins and whole-genome nucleotide sequences consistently showed two groups among 19 *P. gingivalis* strains examined with closely related members: one consisted of ATCC 33277, 381 and HG66, the other of W83, W50 and A7436 [11]. Among them, ATCC 33277 and W83 were mono-clonal but fell in different groups. Obviously, other factors than just clonality affect the virulence of *P. gingivalis* strains. Comparative functional genetics showed strain difference in gingipains, attachment, heme, gene mobility, transposases, capsules, CRISPR and phage [11].

**Figure 2.**
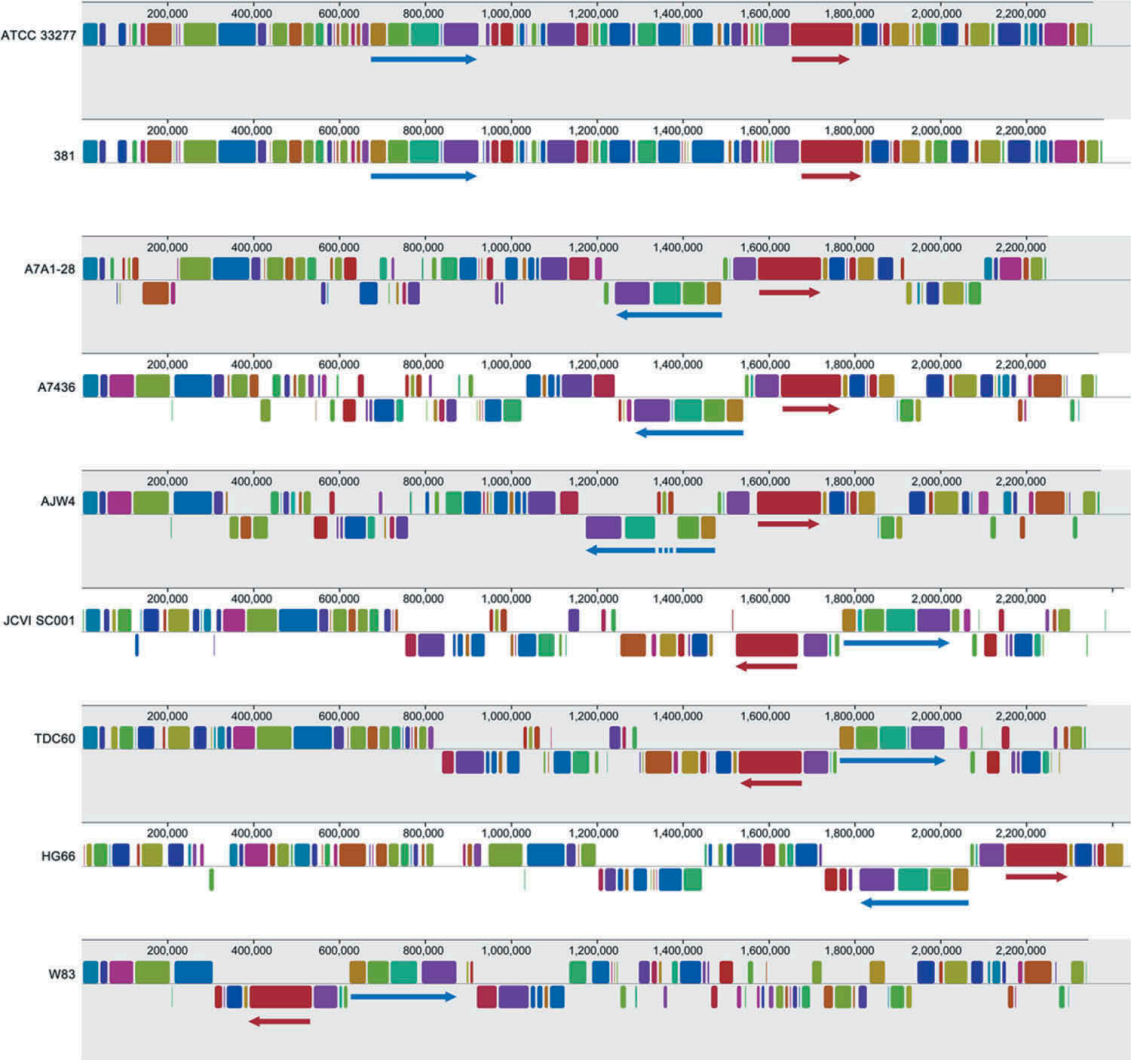
Syntenic analysis of 9 P. *gingivalis* genomic sequences. Of the 35 currently available P. *gingivalis* genomics sequences, eight are considered finished with one final successfully assembled contig. The sequence of JCVI SC001 appears to have a one-contig circular sequence under the Genbank Accession number CM001843, however it is a pseudo-contig generated by ordering the 284 unassembled contigs using the sequence of strain TDC60 as the template. Thus, the syntenies, illustrated as the same color across different genomes, of JCVI SC001 appear to be in similar order of those in TDC60 and may not be the true order in this genome. Syntenies were detected with the “MUAVE” program (version 2.4.0) [57] and were illustrated as rectangles of the same colors across all genomes. Two groups of syntenies were indicated with blue and red arrows underneath to exemplify the different rearrangement and inversion events in different genomes for this species.

Figure 2 shows the homologous DNA segments (syntenies) among the 10 completed *P. gingivalis* genomic sequences. Strains ATCC 33277 and 381 have been shown to be very close and showed the highest similarity between the syntenies detected (i.e. the coloured rectangles in Figure 2 show nearly identical sizes and genomic placement between these two strains). This is as expected. The order and direction of the syntenies indicated the rearrangement of genomic loci, possibly the result of transposition activities in the past. The blue and red arrowhead lines depicted in Figure 2 exemplify the inversion of two regions of syntenies across all genomes. Interestingly, in the genome of AJW4, an insertion event can be observed in the synteny region indicated by the dash part of the blue arrowhead line. These syntenies represent the core genomic loci that are characteristics for this species while the gaps may have been introduced at different times in different environments.

### Insertion segments

Watanabe et al. [42] claimed that the high interspecies diversity in *P. gingivalis* was caused by frequent insertion sequences (ISs). Frandsen et al. [29] found IS*1598* in 69% of 132 *P. gingivalis* strains examined. Altogether, *P. gingivalis* has seven different IS elements named IS*Pg* 1–7 [20]. IS*Pg*5 and other IS elements were proposed to contribute to strain diversity in *P. gingivalis* [36] and they may be used for fingerprinting of strains. More than one IS*Pg* element exists in most strains of *P. gingivalis* and they can probably be active *in vivo* [20]. Examples of IS activity related to pathogenicity include transposition [43], activation of antibiotic resistance genes [43–45], movement and dissemination of virulence genes [46,47], and their inactivation [48–50]. IS transposition can be limited by CRISPR interference as CRISPR limits both IS transposition and the introduction of DNA from other *P. gingivalis* cells [51]. Thus specific domain rearrangements and genetic exchange to generate diversity in surface virulence factors [8] cannot alone be held responsible for the diversity in virulence, although they probably play an important role.

### Plasmid mobilization

CTns are able to mobilize plasmids and other transposons in *Bacteroides*. Natural plasmids have not been detected in *P*. (previously *Bacteroides*) *gingivalis*. However, plasmid and transposon DNA can be conjugally transferred into *P. gingivalis* [52]. This does not apply to *P. gingivalis* strains in general since only some stains could transfer CTns and other accessory mobile elements, while others could not [20]. Thus, plasmid transfer was not possible in W83, possibly because its *traAQ* locus lacks a *traP* homolog. This gene is required for plasmid conjugation in *Bacteroides* [20].

### Concluding remarks

As reviewed, *P. gingivalis* is a species of low clonality that apparently is the result of many accessory genes that contribute to the genomic fluidity, such as the CTns and IS elements. On the other hand, genes that limit the uptake of foreign DNA, e.g. the CRISPR-Cas, are also prevalent in this species. This ‘checks and balances’ governed by the two systems may also be another key to the role of this species in shaping the oral environment in between homeostasis and dysbiosis. It seems possible that genetic transfer and rearrangement makes *P. gingivalis* adapt to and persist in the host environment and that minor changes in the genetic content can cause significant changes in its phenotype. This may be important for the bacterium’s transfer from commensalism to pathogenicity and provides the strains with unique adaptability *in vivo*. Even restricted changes in a single gene may result in extensive biological changes important to the survival of *P. gingivalis* in the host.
